# Personal

**Published:** 1916-01

**Authors:** 


### PERSONAL.

**Announcement of removal, travel, and other matters of interest are requested. Please report errors in the listing of any physician in the State and other directories, that we may co-operate with the State Society in securing a correct list.**

---

Dr. Wesley M. Baldwin has resigned the assistant professorship of anatomy at Cornell and has accepted the corresponding full professorship at the Albany Medical College.

---

Dr. Harry R. Trick of Buffalo made a trip to New York in December.

Dr. B. F. Rogers of Buffalo has gone to California to spend the winter.

---

Miss Ida Caldwell of Buffalo, who has been a visiting nurse, has been engaged in the same capacity for Tonawanda.

---

Dr. J. J. A. McMullen of the Navy Recruiting Station at Buffalo, will be transferred to the Philippines January 18. He will be succeeded by Dr. Harry L. Brown, at present on the U. S. S. Chester.

---

Dr. Willis G. Gregory of Buffalo was the guest of honor at a dinner at the Lafayette, December 8, in commemoration of his 25 years of service as Dean of the Dept. of Pharmacy of the University of Buffalo. The dinner was given by the associated faculties of the University. Dr. Thomas H. McKee, Dean of the Medical Dept. presided. Toast were responded to by Chancellor Chas. P. Norton, Dr. Chas. G. Stockton, Prof. Philip Becker Goetz, Rev. Carl D. Case, D. D., Dean Carlos C. Alden of the Law Dept., Dean Daniel H. Squire of the Dental Dept., Hon. Arthur W. Hickman, Dr. John R. Gray and Dr. Jas. A. Gibson. Dr. A. P. Sy read letters from those unable to be present. Prof. Julian Park of the Arts Dept. presented Dr. Gregory with a clock from the members of the faculties.

---

Dr. Jacob Miller has removed his office from 992 Humboldt Parkway to 558 Ellicott Square, and will limit his practice to Diseases of the Rectum.

